# Role of FNA and Core Biopsy of Primary and Metastatic Liver Disease

**DOI:** 10.1155/2013/174103

**Published:** 2013-11-21

**Authors:** John P. McGahan, John Bishop, John Webb, Lydia Howell, Natalie Torok, Ramit Lamba, Michael T. Corwin

**Affiliations:** ^1^Davis Medical Center, Department of Radiology, University of California, 4860 Y Street, Suite 3100, Sacramento, CA 95817, USA; ^2^Davis Medical Center, Department of Pathology, University of California, 4400 V Street, Path Building, Sacramento, CA 95817, USA; ^3^Davis Medical Center, Department of Internal Medicine, University of California, 4150 V Street, Suite 3500, Sacramento, CA 95817, USA

## Abstract

*Objective*. To examine our experience with cytology and histology biopsy of the liver and to define methods for improvement of diagnosis of primary liver tumors. *Methods*. This include retrospective study of 189 biopsies of 185 liver masses for cytological or histological analysis. Patients were subdivided into two groups. Group 1 consisted of 124 suspected metastasis. Group 2 consisted of 61 suspected primary neoplasms. Biopsies were considered positive or equivocal. In equivocal cases, special stains were performed. In Group 2, cases were classified by contrast CT or MRI as to (I) classic HCC, (II) infiltrated HCC, or (Ill) equivocal. *Results*. Definitive diagnosis was obtained in 117/124 masses (94%) in Group 1, 48/61 masses (79%) in Group 2, and (Ill) equivocal 13 cases in Group II. In two equivocal cases in which special stains were performed, they were reclassified as HCC. In 8/13 cases, CT findings were consistent with HCC. *Conclusion*. Liver biopsies are useful in obtaining a definitive diagnosis of suspected metastatic liver disease. Biopsy results are less reliable in patients with suspected primary liver tumors. In these situations, strategies can include basing treatment on imaging criteria or use of newer special pathological stains. *Advances in Knowledge*. Use of newer special immunological stains improves accuracy in definitive diagnosis of primary liver tumors.

## 1. Introduction

Percutaneous fine needle aspiration cytology (FNAC) and automated needle core biopsy (NCB) for histological retrieval have been used to diagnose malignancy in abdominal organs for over three decades [1, 2]. These techniques of FNAC and NCB have been useful in obtaining a diagnosis of focal liver masses [3–5]. In the past when previously performing liver biopsies of focal masses the biopsies often were performed for metastatic disease. However, more recently we have noticed an increasing number of biopsies for suspected hepatocellular carcinoma. The epidemiology of hepatocellular carcinoma (HCC) is changing in North America and Europe for several reasons, including the viral hepatitis epidemic and the increasing number of patients diagnosed with nonalcoholic fatty liver disease [6]. In these groups, surveillance is difficult and has included use of serum markers such as alpha feto protein (AFP) and imaging with ultrasound, CT, or MRI. However, definitive diagnosis often requires biopsy of focal masses. We have felt that we had maintained very high rate of specific diagnosis for fine needle aspiration cytology (FNAC) or needle core biopsy (NCB) for focal liver masses for possible metastatic disease but have noticed more equivocal results for FNAC or NCB for patients with suspected primary liver tumors. The image guided biopsy strategies are only effective in achieving the goal of reducing mortality if HCC can be adequately distinguished from regenerating nodules by cytology or histology. Our general impression is that definitive diagnosis of HCC is not as easily made as metastatic carcinoma. Therefore, the purpose of this paper is to examine our experience with cytology and histology to find key aspects for the improvement of diagnosis of primary liver tumors, that may be helpful for physicians that encounter patients with suspected primary liver tumors. 

## 2. Materials and Methods

This is a retrospective study conducted at our institution between July 8, 2005 and December 17, 2009. This has full Institution Review Board approval at our institution. Written informed consent was waived. This data set consisted of 240 consecutive targeted liver biopsies performed at our institution. To qualify for the study, a patient had to be over age 18 years and must have a contrast enhanced cross-sectional imaging, magnetic resonance imaging (MRI), or computed tomography (CT) for evaluation of a suspected liver tumor. Computed Tomography was performed with either a General Electric (16 Detector) high speed (General Electric, Milwaukee, WI) or a Siemens Somotom Definition Dual Source (64 Detector) CT (Siemens Medical Solutions USA, Malvern, PA). This included a 4 phase CT including base, arterial phase (approximately 40 seconds), portal venous phase (approximately 90 seconds), and delayed images (3 to 5 minutes) after injection of contrast. Contrast was Omnipaque 350 (GE Healthcare, Princeton, NJ) injected at rate of 3 to 5 mL/second for a total of 125 mL using a power injector. Images were acquired on a 1.5 Tesla General Electric Signa MRI Scanner system (GE Medical Systems, Milwaukee, WI), equipped with a phased array torso coil for signal reception. The imaging protocol was as follows: coronal single shot fast spin echo (SSFSE) transverse SSFSE, transverse noncontrast, T1-weighted, 2D spoiled gradient echo sequence in-phase and out-of-phase, 3D fast relaxation fast spin echo coronal T2-weighted MRCP, 2D thick slab T2-weighted MRCP, and transverse precontrast T1-weighted 3D spoiled gradient echo pulse *(LAVA)* sequence. Then, usually 20 mL of Omniscan (GE Medical Systems, Milwaukee, WI) was injected intravenously at 2 cc/sec and transverse LAVA sequences were obtained with the same parameters as above during the arterial, portal venous, and equilibrium phases. Patients referred to our institution for biopsy with outside imaging were included in the study if their images were stored, deemed adequate, and available on our Picture Archiving and Communications (PAC) system. (I-Site, Koninklijke Philips Electronics, N.V., Foster City, CA).

Twenty-nine patients were excluded from our study since their imaging studies were not available on our PACs system. Fifteen additional patients were excluded as needle aspiration yielded a fluid collection or potential abscess. Since this study addressed percutaneous fine needle aspiration cytology (FNAC) biopsies of liver masses only, six patients whose biopsies were performed under endoscopic ultrasound guidance and one percutaneous biopsy of a bile duct were excluded. 189 liver biopsies which included either FNAC for cytological diagnosis or needle core biopsy (NCB) of the liver for histological diagnosis and or both were obtained. In two cases the masses were rebiopsied and in one case, the mass was biopsied three times, and thus 185 masses were included as the data set in this study. All fine needle aspirations were performed in a similar of fashion using a 22-gauge Chiba type needle with biopsy performed either with an aspiration or non-aspiration technique. At the discretion of the operator. The majority of biopsies are performed under ultrasound guidance with needle visualization documentation in the mass. 

A cytotechnologist was present in the radiology suite to collect the FNAC and NCB specimens. Two slides were prepared from each pass using the “pull-apart method”. One specimen was air dried and the other was fixed in alcohol and immediately evaluated for adequacy by the cytotechnologist following staining with toluidine blue. The air-dried smears were later stained with a modified Giemsa stain in the laboratory and the toluidine blue stained slides were re-strained with a Papanicolaou stain; both types of preparations were evaluated for final diagnosis. The needle from each pass was rinsed in Saccomanno fixative for cell block preparation and reviewed in conjunction with the direct smears for final adequacy determination and final diagnosis. Continued needle passes were performed until cytological technologist confirmed adequacy of the specimen. For NCB, touch prep technique was used in a similar fashion.

Furthermore, liver needle core biopsies (NCB) using an automated biopsy gun or biopsy device were performed with a number of different manufacturers automated devices. Most commonly utilized are automatic 18-gauge core biopsy needle. All NCBs were performed in a similar fashion using ultrasound or CT guidance to document needle position within the lesion. NCBs were collected into formalin, processed and sectioned in the usual fashion, and stained with hematoxylin and eosin. All percutaneous liver biopsies were performed under conscious sedation usually using midazolam hydrochloride (Versed. Hoffman-La Roche) and fentanyl citrate. Study patients were subdivided into two groups based on the clinical and radiologic impression. Group I consisted of 124 biopsies (FNAC and/or NCB) in 124 masses (from 59 men and 65 women; average age 60.3 years) with a suspected diagnosis of hepatic metastases. 

In Group I, if the final cytology or histology report was “adenocarcinoma, site unspecified, this was not considered as a specific diagnosis but was considered as a malignant diagnosis. Group II consisted of 65 biopsies in 61 masses from 61 patients (34 men and 27 women; average age of 58) with a suspected diagnosis of primary liver tumor. We had 185 masses in our database. For those with rebiopsy, only the most definitive biopsy result was included. Forty-nine of sixty one (80%) of Group II patients had underlying cirrhosis which was secondary to hepatitis B or C in 38**  **patients, alcohol-related disease in six, NASH in two, and HCV/HIV in two and autoimmune hepatitis in one. Twelve patients in Group II had no history of hepatitis or cirrhosis. In both groups, the cytology and pathology reports were obtained from the electronic medical record. These results were separated into cases in which a specific diagnosis of a neoplasm or benign entity was made. In Group II, there were 10 reports where the final diagnosis stated, “suspicious for hepatocellular carcinoma.” These were considered as specific diagnoses. When equivocal cytology and pathology results were encountered in Group II, the specific reports and slides were reviewed and classified as 1 = atypical hepatocyte, 2 = HCC versus METS, 3 = HCC versus regenerating nodules, 4 = HCC versus adenoma, 5 = inadequate, 6 = abscess, and 7 = No malignancy. If core biopsy or FNAC was performed on different dates then each report was listed separately.

 Slides from FNAC and NCB cases for patients in Group II with equivocal diagnoses were rereviewed by a cytopathologist (LH) with more than 20 years of experience and a pathologist (JB) with more than 25 years of experience of the FNAC (LH) and the NCB specimens (JB), respectively. They reviewed for any possible misinterpretation by the original pathologist [6]. Furthermore, cell block or core material was applied if possible for immunohistological stains for HEPPAR-1 and glypican-3 [7]. 

The prebiopsy CT or MRI from all available cases in Group II was re-analyzed by a single radiologist (JPM) with more than 25 years of experience. They were classified as pattern I (Focal HCC)—an arterially enhanced mass with a well defined capsule and washout of contrast on delayed imaging in a patient with chronic hepatitis. This includes modification of prior imaging criteria against a background of chronic liver disease (cirrhosis) [8–10]. This also corresponded to the liver imaging and reporting data system (LI-RADS) of definitive HCC [11]. Pattern II consisted of a diffuse irregular enhancing mass with hypervascular portal vein invasion in a patient with hepatitis and chronic liver disease. This corresponded to a LI-RADs category 5V, definitive HCC. Pattern III was considered equivocal for diagnosis based upon lack of pattern I or pattern II. None of the masses were considered definitely benign. 

## 3. Results

Overall, a definitive diagnosis of malignancy was rendered in 165/185 cases (89.1%) based on findings from FNAC and/or NCB results and included both Group I and Group II patients (Table 1). However, when considered separately, Group I and Group II demonstrated differences in the percentage of definitive diagnoses. A diagnosis of malignancy or benign was rendered more frequently in cases where metastatic disease was suspected (94%) compared to cases where hepatocellular carcinoma was suspected (79%). In 117 of 124 (94%) in Group I had a definitive malignant versus benign diagnosis. Core biopsy also more frequently demonstrated definitive malignant versus benign diagnosis than FNA in Group I in 48 of the 50 cases (96%). Furthermore, in the 2 core biopsies in this group that were equivocal, the FNAC results were diagnostic. Therefore 50 of 50 (100%) of those masses with suspected metastatic disease had a definitive malignant versus benign diagnosis by either FNAC or NCB. In Group II, 48/61 masses (79%) had a definitive malignant versus benign diagnosis. In Group II, core biopsy improved detection of primary malignant disease in only 5 additional patients.

CT and MRI were reviewed 54/61 in Group 2 cases and classified as pattern I, pattern II, or pattern III (seven CTs were not retrievable). Pattern I of a classic HCC was seen in 27 of 54 (50%) of cases. Pattern II of an infiltrating tumor and portal vein thrombosis was seen in 8 of 54 (15%) of cases. Pattern III of an equivocal CTs or MRIs was seen in 19 of 54 (35%) of cases. In 14 of 19 equivocal CTs or MRIs cytology/pathology was helpful for giving a definitive diagnosis. 

**Figure 1:**
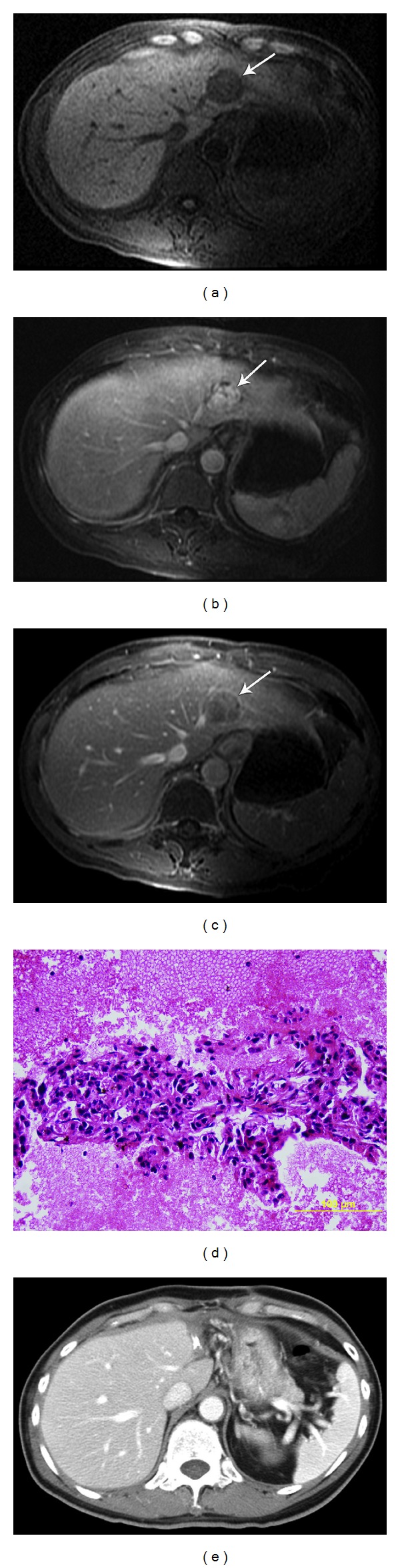
Case  5. History: 60 year-old male with cirrhosis due to hepatitis B with AFP of four. Surgically resected HCCs. Initial MRI demonstrates the following. (a) TI weighted fat suppressed LAVA base scan shows region of decreased signal intensity (arrow). (b) Post gadolinium enhanced MRI in arterial phase shows encapsulated enhancing mass in the left in the lobe of the liver (arrow). (c) After gadolinium in portal venous phase, there is a central wash-out with well-defined capsule (arrow). (d) Fine needle aspiration was deemed suspicious for well differentiated HCC but could not exclude adenoma. The cell block shows groups of hepatocytes forming a thickened trabecular pattern lined by endothelial cells. The hepatocytes have increased nuclear/cytoplasmic (N : C) ratio and occasional prominent nucleoli. (e) After resection of the mass the CT shows surgical clips. Pathology revealed well differentiated HCC.

**Figure 2:**
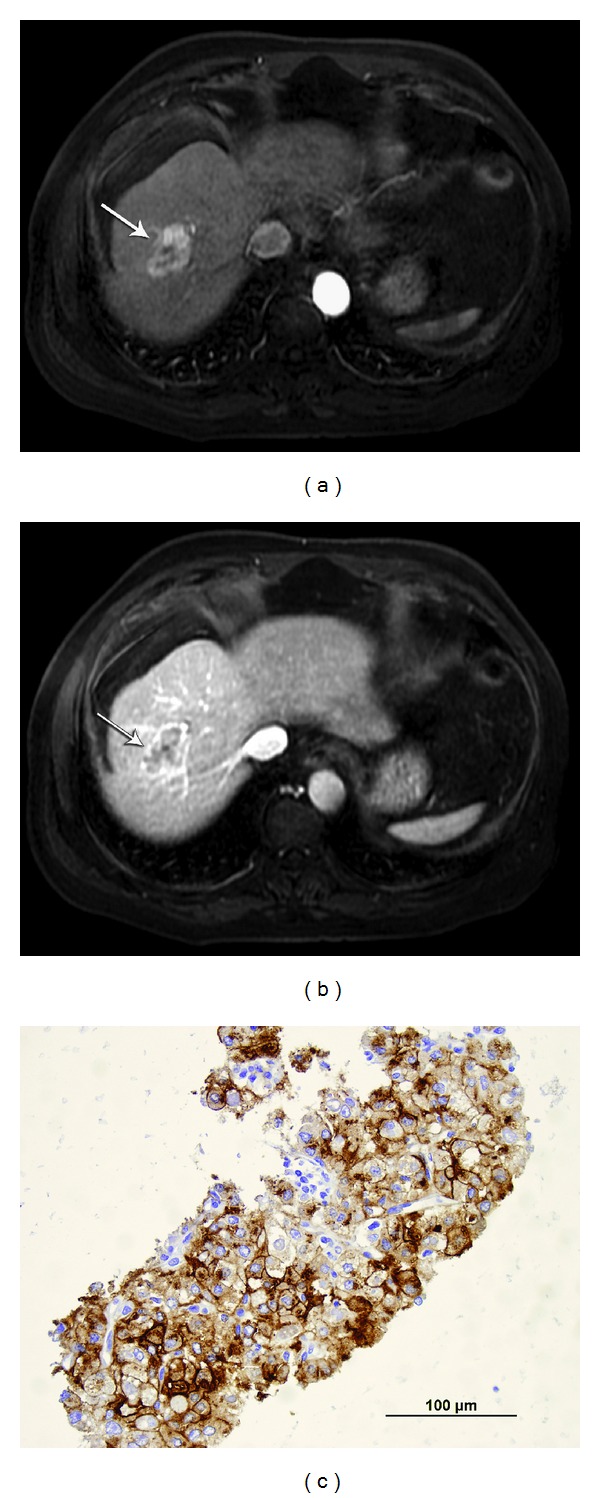
Case  11. History: 61 year-old-male  with AFP of 149 with alcoholic cirrhosis and HCC after transplant. (a) Arterial phase MRI, postgadolinium LAVA sequence showing arterial enhancing lesion (arrow). (b) Post gadolinium MRI during the portal venous phase showed central washout with well defined capsule (arrow). (c) Fine needle aspiration and core were obtained. Fine needle aspiration was suspicious for well differentiated HCC but could not exclude adenoma. Deeper sections of the cell block material revealed acinar-like and branched trabecular patterns of hepatocytes as well as an altered reticulin pattern (not shown). Based on the deeper sections and the positive IHC stain results for glypican-3 (shown here) and Hep Par 1 (not shown), the biopsy is consistent with hepatocellular carcinoma, well-differentiated. Patient had RFA and then liver transplant which revealed HCC.

**Figure 3:**
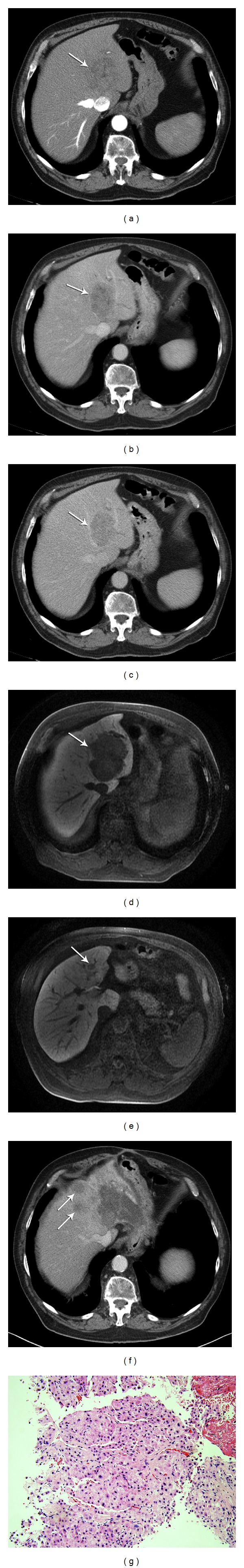
Case  8. History: 72 year-old-male with cirrhosis due to alcoholism with AFP of five. Patient had surgical wedge resection with findings consistent with HCC. (a) Initial arterial CT phase shows encapsulated mass with minimal enhancement (arrow). (b) Portal phase shows well-demarcated area of decrease density with peripheral rim (arrow). (c) Delay imaging demonstrating some washout in this lesion (arrow). This was judged as equivocal for HCC on review. At this time, five FNA's and one core sample which were thought to be nondiagnostic of HCC and were thought to be regenerating nodule versus HCC. (d) MRI with Eovist with 30 minutes delay scan demonstrating area with decrease signal intensity (arrow). (e) An addition a satellite lesion (arrow) was identified cephalad to primary lesion. Three cores were performed on the larger mass which were nondiagnostic and showed no malignancy. (f) Two year follow-up CT demonstrating mass which was locally invasive with multiple satellite lesions throughout the liver (arrows). (g) Other five FNA passes and five cores were obtained at that time which were considered to be satisfactory but nondiagnostic for malignancy. Less than 10% of the histology sample contained hepatocytes for evaluation. Some were disposed in nodules with altered reticulin (not shown). The cells have moderate N : C ratios and are not particularly atypical. Surgical wedge resection was performed which revealed HCC.

**Figure 4:**
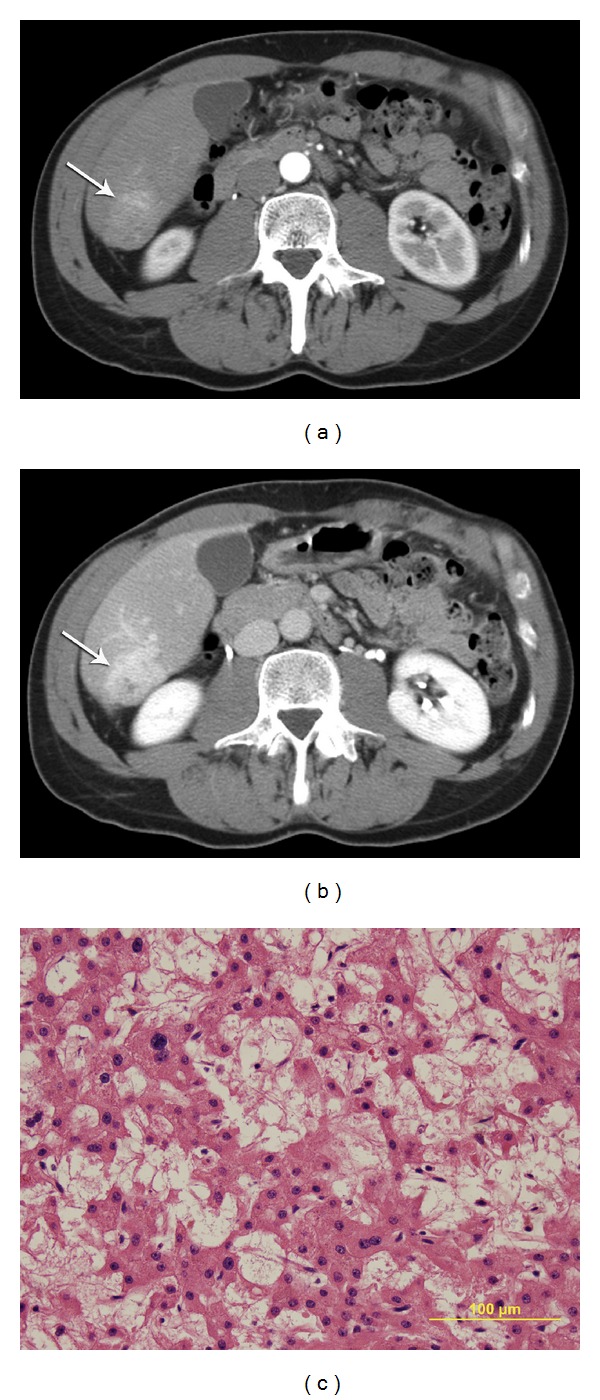
Case  7. History: 51 year-old-male with no cirrhosis, AFP 1.8, and no hepatitis. At surgery this was pathologically proven to represent liver adenoma. (a) Arterial phase CT demonstrating enhancing mass with lobulated margin (arrow). (b) Delay images demonstrating persisting enhancement (arrow) with focal hypodense area noted centrally. This was considered an equivocal mass on review. (c) Fine needle aspiration biopsy with three passes and three core biopsies demonstrated epithelial neoplasm and adenoma versus HCC. This was reviewed at an outside institution with the diagnosis of well-differentiated HCC. The smears are cellular and show hepatocytes with minimal atypia in sheets and small clusters. Prominent capillaries are noted within the sheets and endothelial cells are noted to be encircling clusters of hepatocytes. Post-surgery pathology revealed this to be adenoma.

Furthermore, CT and MRI were analyzed for specific imaging features of HCC in all of the 13 equivocal cytology or pathology of primary liver masses. In 8 of these cases, the radiologic characteristics were nearly pathognomonic for HCC in the setting of cirrhosis (Figures 1 and 2) (Table 2). Six of these cases were classified as pattern I (Classic HCC) with an encapsulated enhancing mass and delayed washout in a cirrhotic liver. Two masses were classified as pattern II with an infiltrating mass and portal vein thrombosis in a cirrhotic liver. 5/13 were equivocal on CT or MRI (Table 2) (Figures 3 and 4). All classic or probable HCCs identified by CT were confirmed by follow-up resection, local progression of disease, or patients expiring with the presumed diagnosis of HCC, except for one case lost to follow-up. Of the 5 equivocal cases on CT or MRI, 1 expired from HCC, 2 had progression of disease, one had a wedge resection revealing HCC and one was an adenoma (Figures 3 and 4). Of the 13 equivocal cytology/histology results, 6 were path proven after operation. Five of these were HCC and one was a hepatic adenoma after operating resection. In 5 cases there was local progression of disease or patients expired, thus with presumed diagnosis of HCC. One is alive and 1 is lost to follow-up (Table 2). The encoded report results from FNA (and core biopsy when performed) of the 13 equivocal cases are also presented in Table 2.

Of these 13 equivocal cytology or pathology cases, six had sufficient cell block or core biopsy material to permit retrospective application of immunohistochemical (IHC) stains for HEPPAR-1 and glypican-3 (Figure 2). The results are shown in Table 3. One case was inconclusive, three were positive only for HEPPAR-1, and two were positive for both antibodies. If positive for both immunohistochemical stains, this is considered to be diagnostic, while if only one immunostain was positive, this was considered as equivocal [7].

## 4. Discussion

Percutaneous fine needle aspiration biopsy of abdominal masses guided by imaging has been performed for over 30 years [2]. With further developments, percutaneous automated biopsy needles have been used for histological retrieval of tissue from abdominal organs [1]. With improved imaging, refinements in biopsy needles, and new pathological techniques, there have been a variety of publications attesting to both high sensitivity and specificity for either fine needle aspiration biopsy or automated needle biopsy of abdominal masses. Numerous publications since the mid-1980s have demonstrated that liver FNAC has sensitivities greater than 85% and a specificity as high as 100%, with the highest sensitivity when FNAC is combined with core biopsy [3–5]. 

As our study demonstrates, FNAC and core biopsy have different limitations prompting equivocal diagnoses. Our findings and other reports have demonstrated that definitive diagnoses are less frequent in cases of hepatocellular carcinoma than metastasis disease. This is true for other primary malignancies, including the thyroid [12, 13]. Reports by both Bru et al. and Matsushiro et al. found that FNAC diagnosis of hepatocellular carcinoma was definitive in only 62% and 61.5% respectively, even when combined with core biopsy [14, 15]. However, others have shown that combining core biopsy with FNAC can improve diagnostic accuracy. Matsushiro et al. demonstrated that adding histological exam via core biopsy improved the positive diagnosis rate to 87% of cases while cytology alone had a positivity rate of 61.5% for the diagnosis of HCC [15]. In our series, we had an overall rate of establishing a diagnosis of 89.1%. (79% for HCC and 94% for suspected metastatic disease, Table 1). So why is there such a discrepancy in establishing a diagnosis of HCC and how can diagnosis accuracy be improved? 

Hepatocellular carcinoma is particularly challenging for both FNA and CB diagnosis. To better understand the challenge, we must understand that all HCCs are not the same. HCC is graded based from I to IV to reflect tumor differentiation and the presence of one or more clonelike cell population [16, 17]. Grade I HCCs can be difficult to distinguish from benign regenerative nodules and adenomas since both show minimal nuclear pleomorphism and prominent nucleoli and have abundant granular cytoplasm reflecting liver differentiation. Architectural features can be the best clues to the diagnosis and include a trabecular pattern and prominent vascular pattern which can be visible on both cytologic smear and core biopsies [18, 19]. This creates a problem for diagnosis. Grade IV HCCs are very poorly differentiated and may be difficult to distinguish from high-grade tumor of non-hepatocellular origin, since features of liver differentiation may not be present [16]. In our series, the ability to distinguish grade I HCC from regenerating nodule or adenomas was the most common reason for lack of definitive diagnosis of HCC by both cytology and histology. Hepatic adenoma was included in the differential diagnosis of the final cytology/pathology report in five masses after either FNAC or core biopsy (Table 2). Three of these patients had cirrhosis. In the other patients with cirrhosis, regenerating nodule versus HCC was mentioned in the final cytology or histology report (Table 2). This diagnostic dilemma may be occurring more frequently due to surveillance programs in patients with underlying liver disease. Not only do these patients have a background of disease with cellular features that can easily mimic malignancy such as balloon degeneration, inflammatory, infiltrate and apoptosis, but the hepatocellular carcinomas targeted for detection are more likely to be welldifferentiated, thus being particularly difficult to distinguish from the background diseased liver. 

How can the problem of distinguishing a grade I HCC from a benign process be addressed?**   **A number of immunohistochemical (IHC) markers have been put forward as possibly helpful in establishing the diagnosis of focal liver lesions. These stains are not be well known to the radiology community, but if requested, they may be useful in establishing or excluding a diagnosis of hepatocellular carcinoma. Markers such Hepatocyte Paraffin Antigen-1 (Hep Par 1), Glypican-3, CD-34, to name a few have been touted as sensitive markers to help distinguish HCC from non HCC malignancy, and are helpful to distinguish Grade 1 HCC form benign liver nodules (Figure 2). Reticulin staining has been traditionally used to enhance the trabecular architecture of primary liver lesions. Immunohistochemical staining with CD-34 may demonstrate diffuse reactivity in HCC and can also be useful [20–24]. It is important for the physicians to know that there are IHC markers that may be helpful when they obtain a biopsy of an equivocal biopsy of potential HCC. Although the best available markers may change, they currently include HepPar-1, glypican-3, and Moc-31, among others [25–27]. The European Association for the study of liver disease published recommendation in 2012 that “immunostaining for GPC3, HSP70, and glutamine synthetase and/or gene expression profiles (GPC3, LYVE1, and survivin) is recommended to differentiate high grade dysplastic nodules from early HCC. Additional staining can be considered to detect progenitor cell features (K19 and EpCAM) or assess neovascularization (CD34)”   [28]. Another recent study has shown that the immunohistochemical panel of Golgi protein 73 (GP73), glypican-3 (GPC 3), and CD 34, as well as reticulum stain, is highly specific in the diagnosis of HCC [29]. However, any of these staining techniques can be performed only on core biopsies (or exceptionally abundant cell blocks) and a sufficiently adequate, representative sample may not be available. In the 13 equivocal cases in our series, there was sufficient material to apply IHC markers in only six cases. Three of those six cases had CT MRI imaging pattern 1 (classic, see Table 3) of which two were positive for both markers and could, in retrospect, be considered as HCC by histopathology, using immunohistochemical markers. However, the other cases were positive for only one marker or were inconclusive (Table 3). Development of molecular tests may also eventually prove useful in distinguishing HCC from reactive disease; however, cost effectiveness will need to be considered when these are developed and implemented since molecular testing is currently very expensive.

What other things may be helpful to establish the diagnosis of HCC if there is an equivocal cytological or histological diagnosis? Rebiopsy with a larger core needle may be considered; low grade HCCs may still be difficult to distinguish from hepatocellular adenomas, as occurred in three of our cases where rebiopsy was performed (Figure 3). While there is poor diagnostic ability of serum AFP to detect HCC [30], serial increase of AFP value clinically may be helpful as a complementary method in chronic hepatitis patients under surveillance for HCC [31]. Thus increasing serum AFP in a cirrhotic patient with a new liver mass with typical features is helpful. More commonly, correlation with imaging findings in cirrhotic patients may play a key role, so that treatment may be undertaken without a definitive diagnosis. The European Association for the study of the liver has states “Noninvasive criteria can only be applied to cirrhotic patients and are based on imaging techniques obtained by 4-phase multidetector CT scan or dynamic contrast-enhanced MRI. Diagnosis should be based on the identification of the typical hallmark of HCC (hypervascular in the arterial phase with washout in the portal venous or delayed phases). While one imaging technique is required for nodules beyond 1 cm in diameter, a more conservative approach with 2 techniques is recommended in suboptimal settings” [28]. The American College of Radiology has also proposed a LI-RADS concept of referring primary liver tumors from LR 1—definitely benign to LR 5—definitely HCC. Thus LR 4 or LR 5 lesions might be considered for definitive treatment as HCC, while LR 3 lesions as intermediate probability may require the potential for biopsy for diagnosis [11]. These are the cases in which liver biopsy may be useful. In our analysis of 54 CTs or MRIs, 19 of the CTs were of pattern III with an equivocal CT diagnosis. Biopsy was definitive of HCC in 14 of 19 of these cases. However, of these 54 cases, the CT or MRI was considered to be of a pattern I or a classic appearance of HCC in 27/54 cases. In 8/54 cases, diffuse tumor with portal venous invasion was also highly suggestive of HCC. In these cases, biopsy could be considered redundant. Of interest in our equivocal biopsy, group of patients with primary liver masses, CT and MRI were very suggestive of a diagnosis in 8 of 13 masses while 5 of 13 were equivocal. Therefore, in the case with equivocal biopsy results, therapy may have to be based upon the aforementioned CT criteria and tumor behavior. Thus, eligibility inclusion criteria for diagnosis and/or treatment could include a positive biopsy with or without immunohistological stain or typical imaging features by CT or MRI in the setting of cirrhosis [11, 30, 32–34]. 

## 5. Limitations

Weakness of this paper includes the fact that this is a retrospective study and that multiple different pathologists were involved in the original interpretation and diagnosis. In addition, because this is a retrospective study, issues that may have limited specimen collection are not known, such as bleeding or technical challenges. 

## 6. Summary

In summary, both percutaneous FNA and NCB demonstrate the ability to differentiate malignant from benign lesions in patients with suspected metastatic liver disease. However, this study confirms that the ability to provide a definitive diagnosis in HCC using FNAC with NCB or alone is more challenging. Using FNAC and NCB for suspected primary liver masses, regenerating nodules, and well-differentiated HCC frequently has overlapping cytologic features. These diagnostic challenges and their subsequent management issues may increase as surveillance of patients with liver disease becomes more commonplace. These strategies can include basing treatment on CT or MRI with specific imaging features in a cirrhotic liver with the appropriate clinical and biochemical setting. Effective management may be based upon not only definitive cytological and histological results, but also the recognition that there are special immunohistochemical stains that may prove useful in distinguishing HCC from reactive disease.

## Figures and Tables

**Table 1:** Results.

	FNA		CB		FNA and CB	
Suspected hepatic metastasis						
Specific diagnosis	56%	(70/124)	68%	(34/50)	60%	(75/124)
^ ∗^Diagnosis malignant versus benign	93%	(115/124)	96%	(48/50)	94%	(117/124)
Suspected primary neoplasms						
Specific diagnosis	70.5%	(43/61)	67.5%	(27/40)	79%	(48/61)

Total	85.4%	(158/185)	83%	(75/90)	89.1%	(165/185)

*Diagnosis of malignancy, but type not specified.

**Table 2:** Features of the equivocal cases.

Case	AFP	Cirrhosis	CT	Diagnosis	FNA	Core
1	6.6	C	I (classic)	RFA-alive	1	None
2	49	B + C	III equivocal	Expired HCC	2	None
3	14.6	C + ETOH	III equivocal	Progression	1, 1	3
4	241	C	I (classic)	Path proven HCC-surgery	3	None
5	4.1	B	I (classic)	Path proven HCC-surgery	4	None
6	3.6	C	I (classic)	Path proven HCC-surgery	6	2
7	1.8	None	III equivocal	Adenoma	4	4
8	5	C	III equivocal	Path proven HCC-surgery	3, 7	7, 7, 7
9	None	ETOH	II infiltration	Died HCC	5	None
10	14,044	C	II infiltration	Hospice HCC	1, 3	7
11	149	ETOH	I (classic)	Path proven HCC-surgery. RFA transplant	4	7
12	6.4	None	III equivocal	Progression	7	4
13	19.5	C	I (classic)	Lost to follow-up	4	7

1: atypical hepatocyte; 2: HCC versus METS; 3: HCC versus regenerating nodules; 4: HCC versus adenoma; 5: inadequate; 6: abscess; 7: no malignancy. Equivocal cases including Alpha Fetoprotein (AFP) values and presence of cirrhosis including etiology for example hepatitis C (C), hepatitis B (B), or alcohol (ETOH). The review of CT results, patient follow-up and classification of results from the cytology or histology report.

**Table 3:** Results of stains applied retrospectively on equivocal biopsies.

Case	CT	Diagnosis	HepPar1	Glypican3
3	III equivocal	Progression	pos	neg
5	I (classic)	Path proven HCC-surgery	Inconclusive	Inconclusive
8	I (classic)	Path proven HCC-surgery	pos	neg
10	II infiltration	Hospice HCC	pos	neg
11	I (classic)	Path proven HCC-surgery. RFA transplant	pos	pos
13	I (classic)	Lost to follow-up	pos	pos

^∗^7 of the 13 equivocal cases did not have sufficient material for these stains.
